# Prediction of lymph node metastasis in cN0 papillary thyroid carcinoma using a random forest–based radiomics model integrating contrast-enhanced ultrasound and clinical data

**DOI:** 10.3389/fonc.2026.1728554

**Published:** 2026-03-23

**Authors:** Shasha Yuan, Di Tang, Lei Han, Yu Sun, Ying Liu

**Affiliations:** Department of Ultrasound, Harbin Medical University Cancer Hospital, Harbin, Heilongjiang, China

**Keywords:** clinical data, contrast-enhanced ultrasound, lymph node metastasis, papillary thyroid carcinoma, radiomics

## Abstract

**Objectives:**

The aim of this study was to evaluate a radiomics model integrating contrast-enhanced ultrasound (CEUS) features and clinical indicators for predicting lymph node metastasis (LNM) in patients with papillary thyroid carcinoma (PTC) classified as clinically node-negative (cN0).

**Methods:**

A total of 604 patients with preoperative cN0 PTC who underwent routine ultrasound and CEUS examinations were enrolled. Radiomics features were extracted from ultrasound images and integrated with contrast parameters and clinical indicators to construct machine learning models. The diagnostic performance of different models in predicting central lymph node metastasis (CLNM) and overall LNM was compared. Feature selection was performed using least absolute shrinkage and selection operator (LASSO) regression analysis. Performance metrics were compared to determine the optimal model. Receiver operating characteristic (ROC) curves, precision–recall (PR) curves, calibration curves, and decision curves were generated to illustrate differences in diagnostic performance. SHapley Additive exPlanations (SHAP) were applied for model interpretability.

**Results:**

Data were divided into five groups. The Clinical + Radiomics + Contrast parameter random forest (RF) model demonstrated the highest predictive performance for both CLNM and LNM. The two models incorporated different predictive indicators. The area under the curve (AUC) values were 0.928 and 0.942, respectively. SHAP visualization identified 20 important features for each optimal model. Radiomics features, particularly those derived from CEUS images, along with contrast parameters, demonstrated significant predictive value in addition to clinical characteristics.

**Conclusions:**

Radiomics features and CEUS contrast parameters enhanced the diagnostic efficacy of predictive models. The RF model integrating clinical, radiomics, and contrast parameter data demonstrated the highest predictive accuracy for CLNM and LNM in patients with cN0 PTC.

## Introduction

Papillary thyroid carcinoma (PTC) represents the most common subtype of thyroid cancer, accounting for more than 80% of all pathological cases (1). The incidence of metastatic lateral cervical lymph nodes (LNs) in PTC ranges from 20% to 50% (2). Ultrasound (US) is the first-line imaging modality for evaluating thyroid and cervical lymph node abnormalities, despite the availability of other methods (3). Contrast-enhanced ultrasound (CEUS) plays a significant role in the evaluation of thyroid nodules and the prediction of cervical lymph node metastasis (LNM). However, preoperative ultrasound detects fewer than 30% of LNMs, a limitation that may be related to tracheal gas interference and operator experience (4). Accurate preoperative LNM prediction is critical for determining the surgical scope and assessing the risk of recurrence and metastasis in PTC (5). Therefore, developing novel predictive model is necessary to improve the accuracy of preoperative evaluation of cervical LNM in PTC.

Radiomics, first proposed by Lambin in 2012 (6), utilizes machine learning techniques to convert medical images into a high-dimensional data space, thereby establishing relationships between imaging characteristics and pathology. This approach underpins clinical decision-making in the evaluation of tumor status (7), cancer grading, and metastasis risk stratification (8). In recent years, radiomics has emerged as an advanced diagnostic technology capable of extracting sub-visual imaging features to support disease diagnosis and prognosis. Intra- and interobserver variability remains a significant limitation in US examinations. By revealing disease patterns not apparent through conventional analysis, artificial intelligence (AI)–based radiomics enables the extraction of high-throughput quantitative features from imaging data (9).

Evidence has indicated that radiomics can predict cervical lymph node metastasis in PTC, particularly metastasis to central lymph nodes. Such predictive capability has the potential to reduce unnecessary central lymph node dissections and to aid in identifying patients suitable for radiofrequency ablation, thereby mitigating overtreatment. However, the predictive capacity of radiomics alone is limited due to the relatively small number of incorporated variables and its reduced accuracy, which has constrained its broader clinical application.

**Figure 1:**
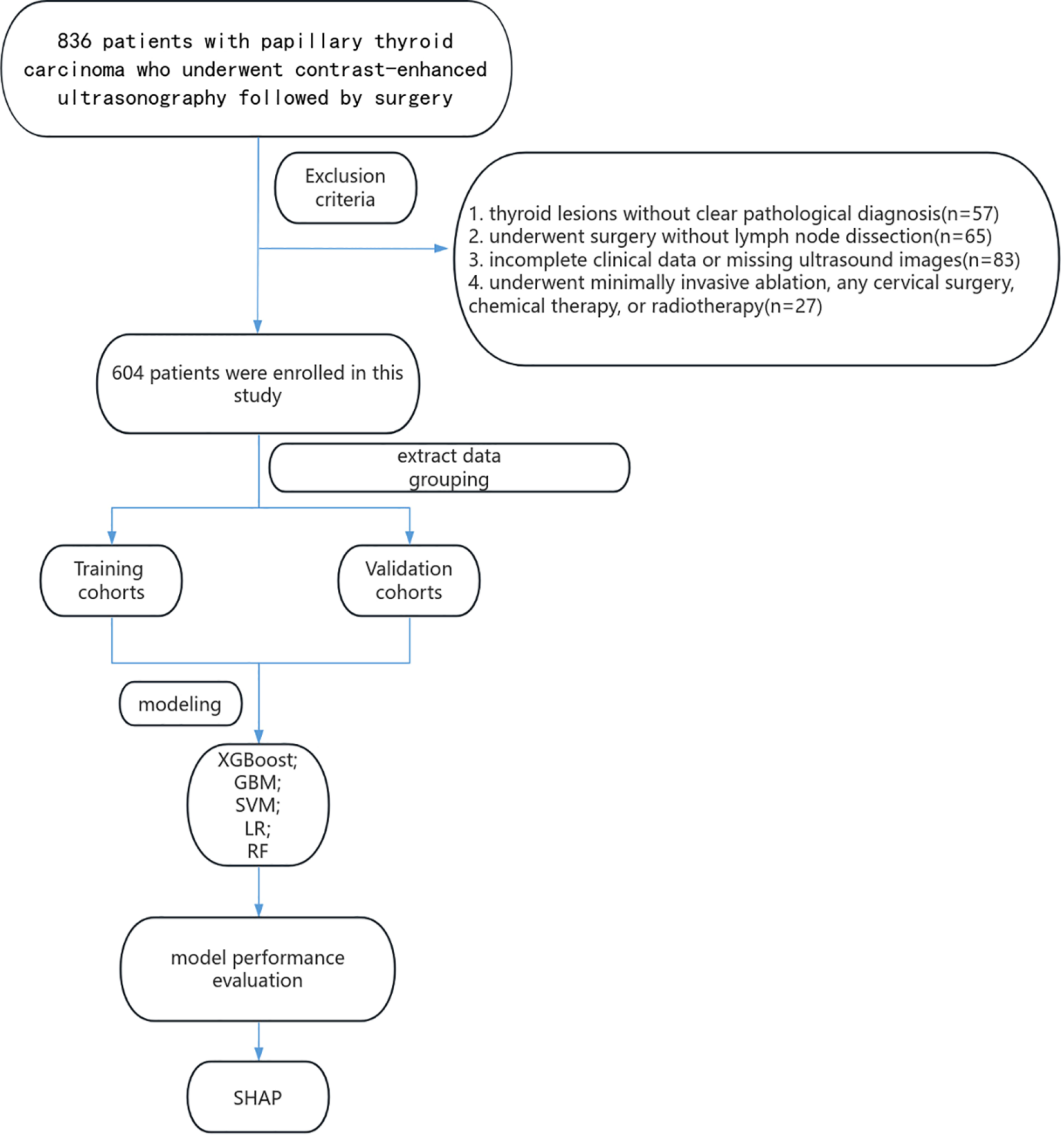
Flow diagram of the study design.

Research on the utility of CEUS for predicting LNM in PTC remains limited. This investigation aimed to develop a novel and reliable predictive model by integrating conventional ultrasound, CEUS, and clinical indicators, with the goal of enhancing the accuracy of preoperative prediction of cervical LNM in PTC and providing new insights for personalized treatment. The study specifically addressed the following objectives: (1) to determine whether radiomics features and CEUS contrast parameters enhance the diagnostic performance of the model, (2) to identify the machine learning approach with the highest predictive ability, and (3) to evaluate whether the optimal model selects distinct features for predicting LNM and central lymph node metastasis (CLNM) (**Figure 1**).

## Materials and methods

### Patients

A total of 836 patients with PTC who underwent contrast-enhanced ultrasonography followed by surgery at Harbin Medical University Cancer Hospital between March 2020 and May 2025 were retrospectively included. All patients were preoperatively diagnosed as having no regional lymph node metastasis (cN0) by ultrasound. Written informed consent was waived for this retrospective analysis.

Inclusion criteria were: (1) postoperative pathology confirming PTC; (2) patients undergoing initial thyroidectomy with lymph node dissection; (3) no history of other malignant tumors; and (4) availability of complete clinical and ultrasound image data.

Exclusion criteria included: (1) thyroid lesions without a definitive pathological diagnosis; (2) surgery performed without lymph node dissection; (3) incomplete clinical data or missing ultrasound images; and (4) patients who had previously undergone minimally invasive ablation, any cervical surgery, chemotherapy, or radiotherapy. Following screening, 604 patients were enrolled and randomly assigned to the training and validation cohorts in a 7:3 ratio.

### Ultrasound image acquisition and delineation of regions of interest

Ultrasound examinations were performed by a radiologist with over 12 years of experience in thyroid ultrasonography. All patients underwent routine ultrasound and CEUS examination. Routine ultrasound was conducted using the SuperSonic Imagine AixPlorer real-time shear wave ultrasound elastography system (France) with a probe frequency of 4–15 MHz and shear wave elastography (SWE) mode. CEUS images were acquired using the Mindray A20 and Risonna 7 systems. The contrast agent SonoVue was dissolved in 5 mL of physiological saline and intravenously injected at a dose of 1.5 mL via the anterior cubital vein for each CEUS examination. Real-time microbubble perfusion in nodules and surrounding tissues was observed for at least two minutes and recorded on the internal hard drive of the ultrasound system. For patients with multiple thyroid nodules, the tumor with the largest diameter on ultrasound was selected for analysis.

**Figure 2:**
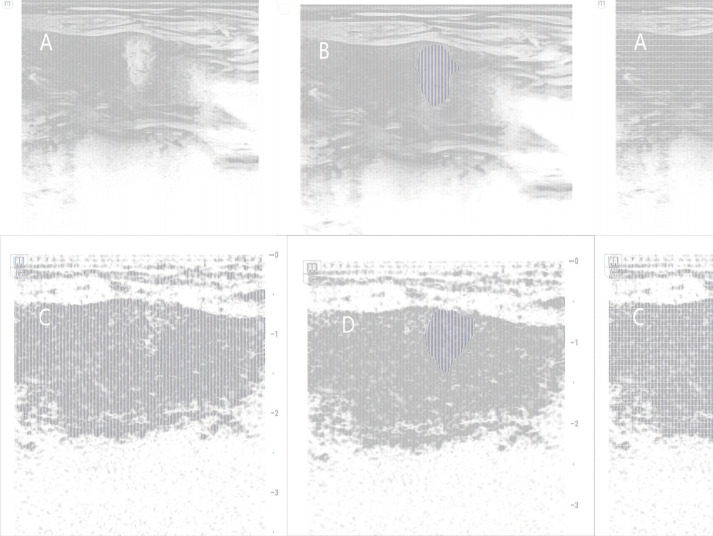
ROI delineation images for B-mode ultrasound and CEUS. **(A, B)** is B-mode ultrasound images. **(C, D)** is CEUS images.

The grayscale ultrasound image of the targeted tumor along its longest axis was selected and labeled as Image 1 for each patient. The CEUS image selected for analysis was captured at the peak contrast agent concentration of the lesion and labeled as Image 2. All images were exported in JPEG format and converted to Neuroimaging Informatics Technology Initiative (NII) format using conversion software. Regions of interest (ROI) were manually delineated by a sonographer with over ten years of thyroid ultrasound experience using the open-source software ITK-SNAP (version 3.8.0; http://www.itksnap.org) (**Figure 2**).

### Research groups

Radiomics data were obtained from ultrasound images, while CEUS parameters were extracted from contrast imaging features, including enhancement mode (uniform, non-uniform, no enhancement, circular enhancement), enhancement degree (no enhancement, high, equal, low enhancement), post-enhancement boundary clarity, membrane continuity (yes/no), and wash-out pattern (early, synchronous, slow).

The collected data were organized into five groups: (1) clinical data; (2) radiomics; (3) clinical data + radiomics; (4) radiomics + contrast parameters; and (5) clinical data + radiomics + contrast parameters. A standalone contrast parameter group was excluded due to the limited number of indicators, which did not support effective model construction. Since contrast parameters are derived from contrast imaging, they were not classified independently with clinical data in the research groups.

### Data preprocessing and radiomics feature screening

Initially, MixUp image enhancement was applied to ultrasound images. Gaussian Laplace (LoG) filters (σ = 1, 3), wavelet filters, square filters, square root filters, exponential filters, and gradient filters were applied sequentially to the enhanced images. Radiomics features from ROIs were extracted using the open-source PyRadiomics toolkit (version 3.0.1; https://github.com/Radiomics/pyradiomics). Non-count features were normalized using min–max scaling. Feature selection was performed using least absolute shrinkage and selection operator (LASSO) regression analysis. Features with non-zero coefficients were retained based on the optimal λ parameter and regularization path, ensuring representativeness and stability of the feature set.

### Model construction

Five machine learning algorithms were evaluated: extreme gradient boosting (XGBoost), gradient boosting machine (GBM), support vector machine (SVM), logistic regression (LR) analysis, and random forest (RF). Hyperparameter optimization was conducted using the Optuna framework to identify the optimal parameter set for each model. Five-fold cross-validation was applied to assess model performance in the training and validation cohorts.

Model performance was evaluated using area under the curve (AUC), accuracy, precision, recall/sensitivity, specificity, F1 score, and Brier score. Receiver operating characteristic (ROC) curves, precision-recall (PR) curves, calibration curves, and decision curves were plotted to visualize diagnostic performance differences. SHapley Additive exPlanations (SHAP) were employed for model interpretability.

### Experimental environment and software dependence

All data processing and model training were performed in a Python (version 3.10) environment. Key toolkits included XGBoost (version 1.7.6; https://github.com/dmlc/xgboost/), GBM (version 4.3.0; https://github.com/microsoft/LightGBM/), scikit-learn (version 1.4.2; https://scikit-learn.org/stable/), Optuna (version 3.6.1; https://optuna.org/), and SHAP (version 0.41.0; https://github.com/slundberg/shap).

### Statistical analysis

Statistical analyses were conducted using the StatsModels package in Python (version 0.14.4; https://www.statsmodels.org/stable/index.html). Continuous variables with normal distribution were compared using the independent sample *t*-test, whereas non-normally distributed variables were compared using the Wilcoxon rank-sum test. Categorical variables were compared using Pearson’s chi-squared test or Fisher’s exact test, with a significance threshold of *p* < 0.05.

## Results

### Clinical features of papillary thyroid carcinoma

A total of 604 patients were included in this study, comprising 95 males and 509 females, with a median age of 44.9 years (range: 14–72 years). Among these, 154 patients presented with CLNM and 163 patients with LNM, including 9 cases of skip metastasis, while 441 patients demonstrated no evidence of metastasis confirmed by postoperative pathology. The training cohort consisted of 442 patients (62 males and 360 females), and the validation cohort included 182 patients (33 males and 149 females). The clinical data collected encompassed demographic information, body measurements, and biochemical markers, including gender, age, height, weight, body mass index (BMI), presence of diffuse thyroid lesions, Thyroid Imaging Reporting and Data System (TIRADS) classification, tumor location, size, ultrasound features (echogenicity, morphology, margins, aspect ratio, presence of microcalcifications, posterior acoustic features, blood flow characteristics), SWE values (Emax, Emean, Emin, elasticity index [EI]), and serum markers (thyroid stimulating hormone [TSH], free thyroxine [FT4], free triiodothyronine [FT3], thyroglobulin antibodies [Anti-TG], thrombomodulin antibody [Anti-TM], thyroglobulin [Tg], parathormone [PTH], hematocrit [hCT]), as well as BRAF V600E mutation status. Clinical characteristics with significant differences (*p* < 0.05) are presented in **Table 1**.

**Table 1:** Univariate analysis of lateral cervical micro-lymph node metastases of papillary thyroid carcinoma was concentrated in the training and testing sets.

Variables	Training cohorts (N=)			Validation cohorts (N=)			Training cohorts (N=)			Validation cohorts (N=)		
	CLNM (-)	CLNM (+)	*P* value	CLNM (-)	CLNM (+)	*P* value	LNM (-)	LNM (+)	*P* value	LNM (-)	LNM (+)	*P* value
	N=321	N=101		N=129	N=53		N=314	N=108		N=127	N=55	
Gender												
Male	39	23	0.014	19	14	0.099	37	25	0.007	19	14	0.139
Female	282	78		110	39		277	83		108	41	
Age	46.489 (18,71)	40.030 (14,71)	<0.001	46.705 (25,72)	40.604 (27,60)	<0.001	46.713 (18,71)	39.796 (14,71)	<0.001	46.874 (25,72)	40.436 (27,60)	<0.001
Height	1.624 (1.45,1.80)	1.644 (1.36,1.86)	0.025	1.627 (1.475,1.87)	1.639 (1.51,1.80)	0.283	1.624 (1.45,1.80)	1.643 (1.36,1.86)	0.026	1.627 (1.475,1.87)	1.639 (1.51,1.80)	0.274
Max Diameter	7.207 (1.9,30)	9.543 (2.9,34)	<0.001	7.510 (2.7,22)	9.157 (2.7,35)	0.046	7.145 (1.9,30)	9.571 (2.9,34)	<0.001	7.471 (2.7,22)	9.187 (2.7,35)	0.034
Horizontal Diameter	6.743 (1.4,30)	9.231 (2,34)	<0.001	7.024 (1.8,22)	8.749 (2.5,35)	0.045	6.675 (1.4,30)	9.268 (2,34)	<0.001	6.977 (1.8,22)	8.795 (2.5,35)	0.031
Vertical Diameter	5.880 (1.9,17)	7.177 (2.9,25)	0.001	7.024 (1.8,22)	8.749 (2.5,35)	0.045	5.845 (1.9,17)	7.194 (2,25)	<0.001	6.977 (1.8,22)	8.795 (2.5,35)	0.031
Aspect Ratio												
≤1	164	66	0.017	66	32	0.332	160	70	0.017	64	34	0.208
>1	157	35		63	21		154	38		63	21	
Echotexture												
Uniform	145	28	0.003	59	15	0.044	144	29	0.001	59	15	0.024
Not uniform	176	73		70	38		170	79		68	40	
Microcalcifications												
No	147	28	0.004	60	10	0.001	146	29	0.001	60	10	0.000
Yes	174	73		69	43		168	79		67	45	
Shape												
Regular	285	79	0.012	117	40	0.013	279	85	0.013	116	41	0.005
Irregular	36	22		12	13		35	23		11	14	

### Radiomics characteristic data

A total of 936 radiomics features were extracted from conventional ultrasound and CEUS images for analysis. Feature selection was performed using LASSO regression analysis, with features meeting selection criteria retained for model construction. The radiomics features selected varied across different research groups due to the distinct modeling strategies.

### Characteristics of contrast parameters

Among all patients, 401 exhibited unclear boundaries after enhancement, and 84 presented with discontinuous capsule integrity. Enhancement patterns included nonuniform enhancement in 589 patients, uniform enhancement in 10, equal enhancement in 26, high enhancement in 35, and low enhancement in 542 patients. Washout patterns included early washout in 515 patients, synchronous washout in 33, and slow washout in 56 patients.

### Evaluation of model performance

**Figure 3:**
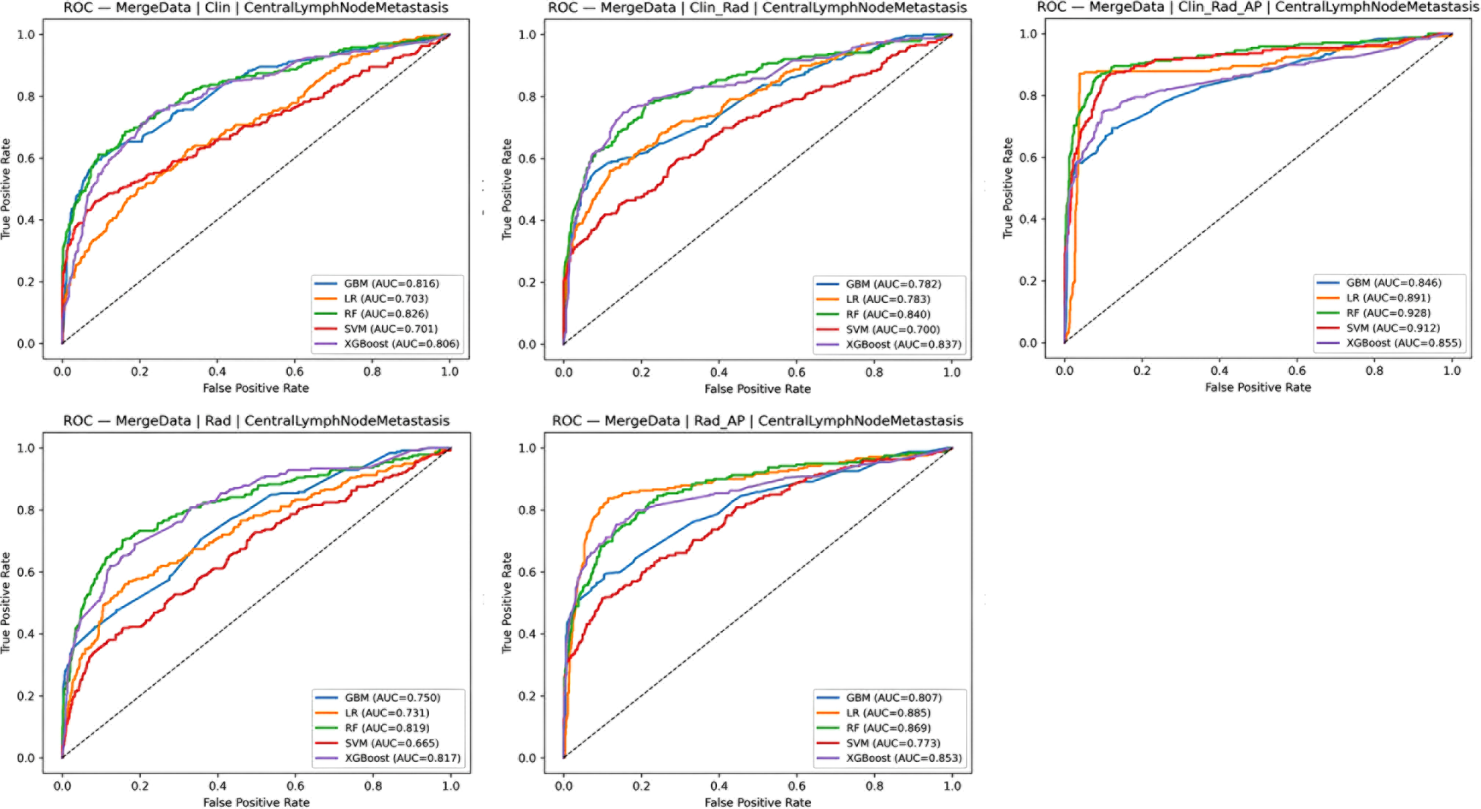
Comparison of ROC in testing sets of each CLNM models.

**Figure 4:**
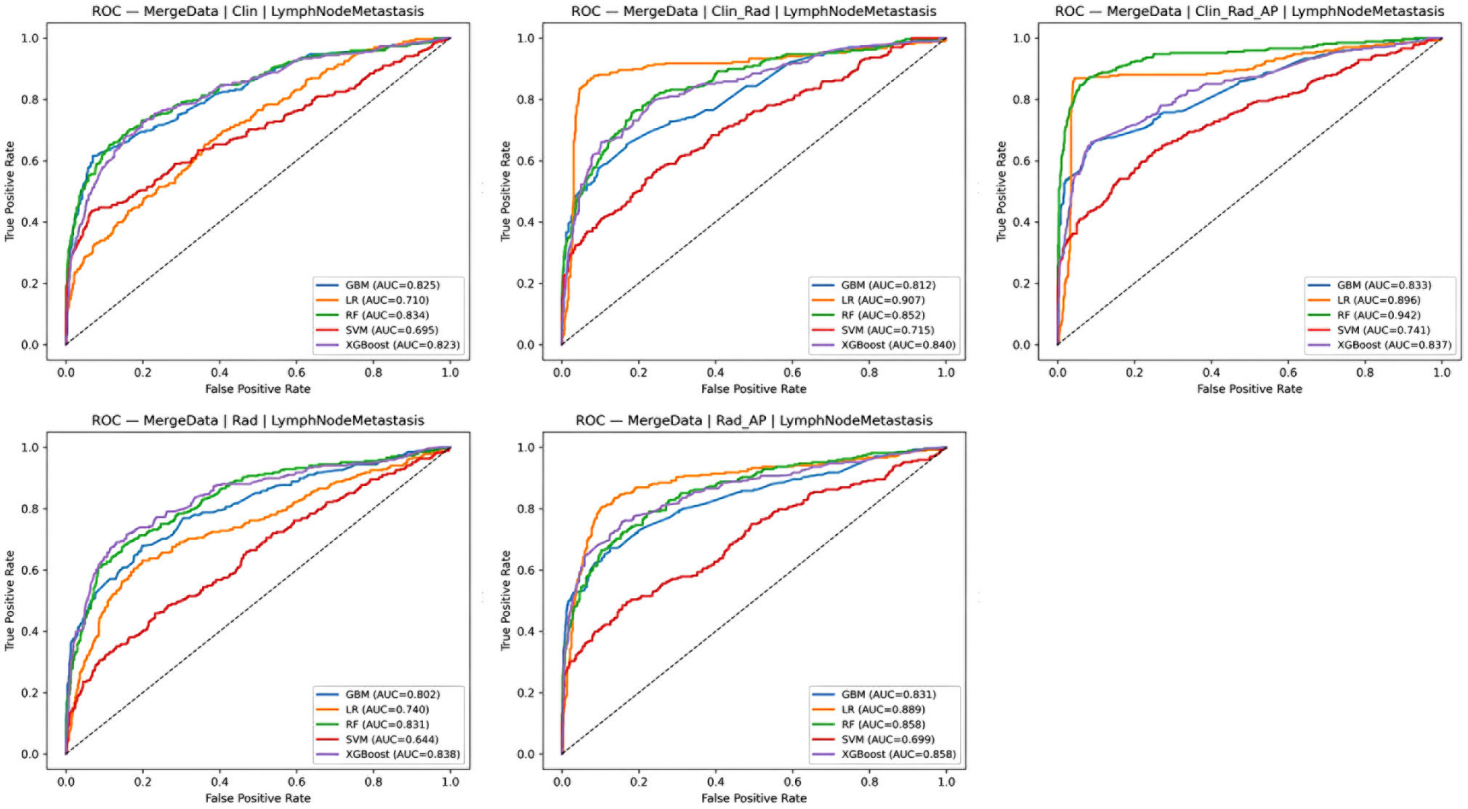
Comparison of ROC in testing sets of each LNM models.

The data were divided into five groups for analysis: (1) clinical data; (2) radiomics; (3) clinical data + radiomics; (4) radiomics + contrast parameters; and (5) clinical data + radiomics + contrast parameters. Five machine learning models—XGBoost, GBM, SVM, LR analysis, and RF—were developed for each group. Model performance in predicting LNM and CLNM was compared, and predictive metrics for each model are summarized in **Tables 2**, **3**. AUC comparisons are presented in **Figures 3**, **4**.

**Table 2:** Comparison of optimal prediction model for each group in CLNM.

Group	Model	AUC	Accuracy	Precision	Specificity	PR_AP	Brier score
Clinical	RF	0.826	0.817	1.000	1.000	0.702	0.136
Radiomics	RF	0.819	0.819	0.869	0.990	0.668	0.133
Radiomics+Contrast parameters	LR	0.885	0.891	0.782	0.943	0.731	0.093
Clinical+Radiomics	RF	0.840	0.823	0.981	0.998	0.710	0.131
Clinical+Radiomics+Contrast parameters	RF	0.928	0.854	0.966	0.996	0.868	0.101

**Table 3:** Comparison of optimal prediction model for each group in LNM.

Group	Model	AUC	Accuracy	Precision	Specificity	PR_AP	Brier score
Clinical	RF	0.834	0.800	0.966	0.997	0.728	0.144
Radiomics	RF	0.831	0.792	0.881	0.991	0.687	0.145
Radiomics+Contrast parameters	LR	0.889	0.874	0.771	0.930	0.756	0.100
Clinical+Radiomics	LR	0.907	0.921	0.843	0.948	0.770	0.078
Clinical+Radiomics+Contrast parameters	RF	0.942	0.836	0.979	0.998	0.901	0.109

For CLNM prediction, the optimal models were: Clinical data group: RF model (AUC = 0.826, PR_AP = 0.702, Brier score = 0.136, accuracy = 0.817, recall = 0.179, F1 = 0.305), radiomics group: RF model (AUC = 0.819, PR_AP = 0.668, Brier score = 0.133, accuracy = 0.819, recall = 0.222, F1 = 0.353), radiomics + contrast parameters group: LR analysis model (AUC = 0.885, PR_AP = 0.731, Brier score = 0.093, accuracy = 0.891, recall = 0.707, F1 = 0.743), clinical data + radiomics group: RF model (AUC = 0.840, PR_AP = 0.710, Brier score = 0.131, accuracy = 0.823, recall = 0.213, F1 = 0.351), clinical data + radiomics + contrast parameters group: RF model (AUC = 0.928, PR_AP = 0.868, Brier score = 0.101, accuracy = 0.854, recall = 0.360, F1 = 0.524).

For LNM prediction, the optimal models were: Clinical data group: RF model (AUC = 0.834, PR_AP = 0.728, Brier score = 0.144, accuracy = 0.800, recall = 0.209, F1 = 0.343), radiomics group: RF model (AUC = 0.831, PR_AP = 0.687, Brier score = 0.145, accuracy = 0.792, recall = 0.194, F1 = 0.318), radiomics + contrast parameters group: LR analysis model (AUC = 0.889, PR_AP = 0.756, Brier score = 0.100, accuracy = 0.874, recall = 0.705, F1 = 0.737), clinical data + radiomics group: LR analysis model (AUC = 0.907, PR_AP = 0.770, Brier score = 0.078, accuracy = 0.921, recall = 0.840, F1 = 0.841), clinical data + radiomics + contrast parameters group: RF model (AUC = 0.942, PR_AP = 0.901, Brier score = 0.109, accuracy = 0.836, recall = 0.354, F1 = 0.521).

### Results of intergroup comparison of optimal models

**Figure 5:**
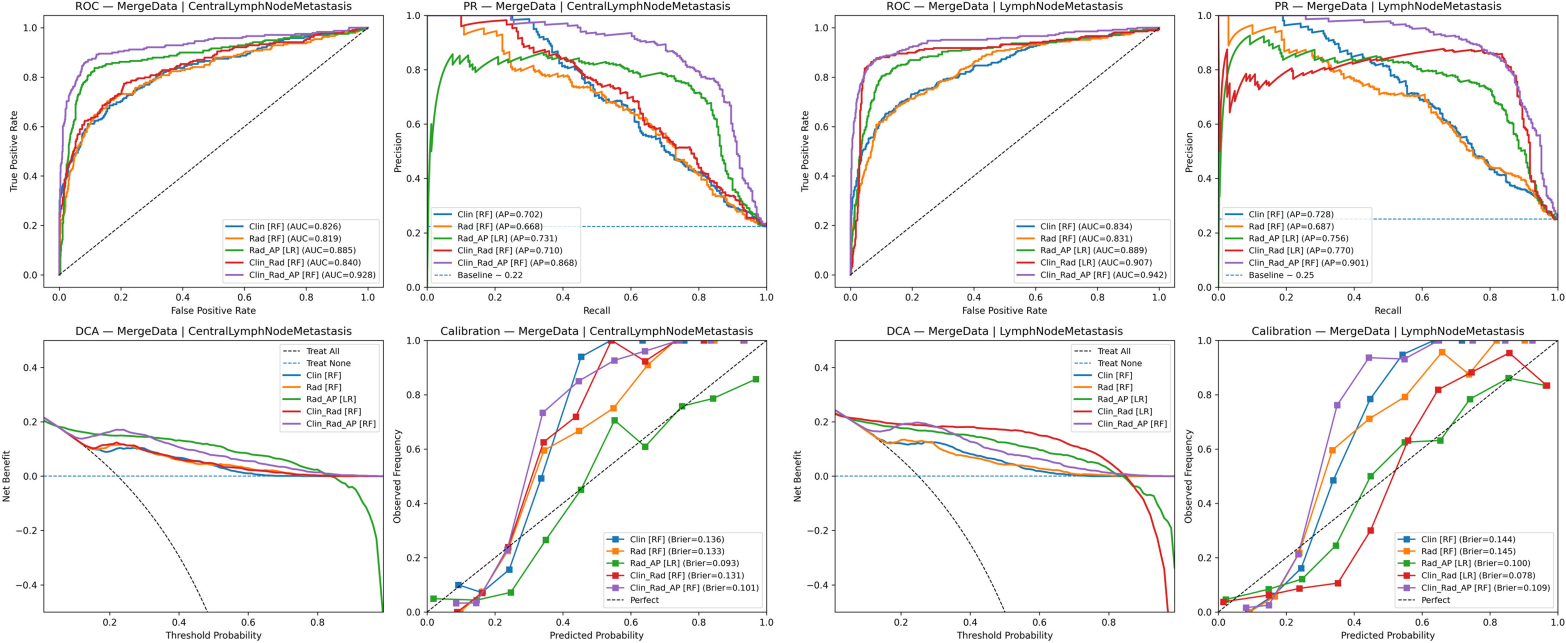
Comparison of optimal model for each group in predicting CLNM and LNM.

Comparative analysis of AUC, precision-recall (PR) curves, decision curve analysis (DCA), and calibration curves of optimal models for each group is presented in **Figure 5**. The clinical data + radiomics + contrast parameters group using the RF model demonstrated the highest performance for both CLNM and LNM prediction, indicating that this combination yields the best predictive capacity.

### SHAP visualization analysis of clinical + radiomics + contrast parameter RF model

**Figure 6:**
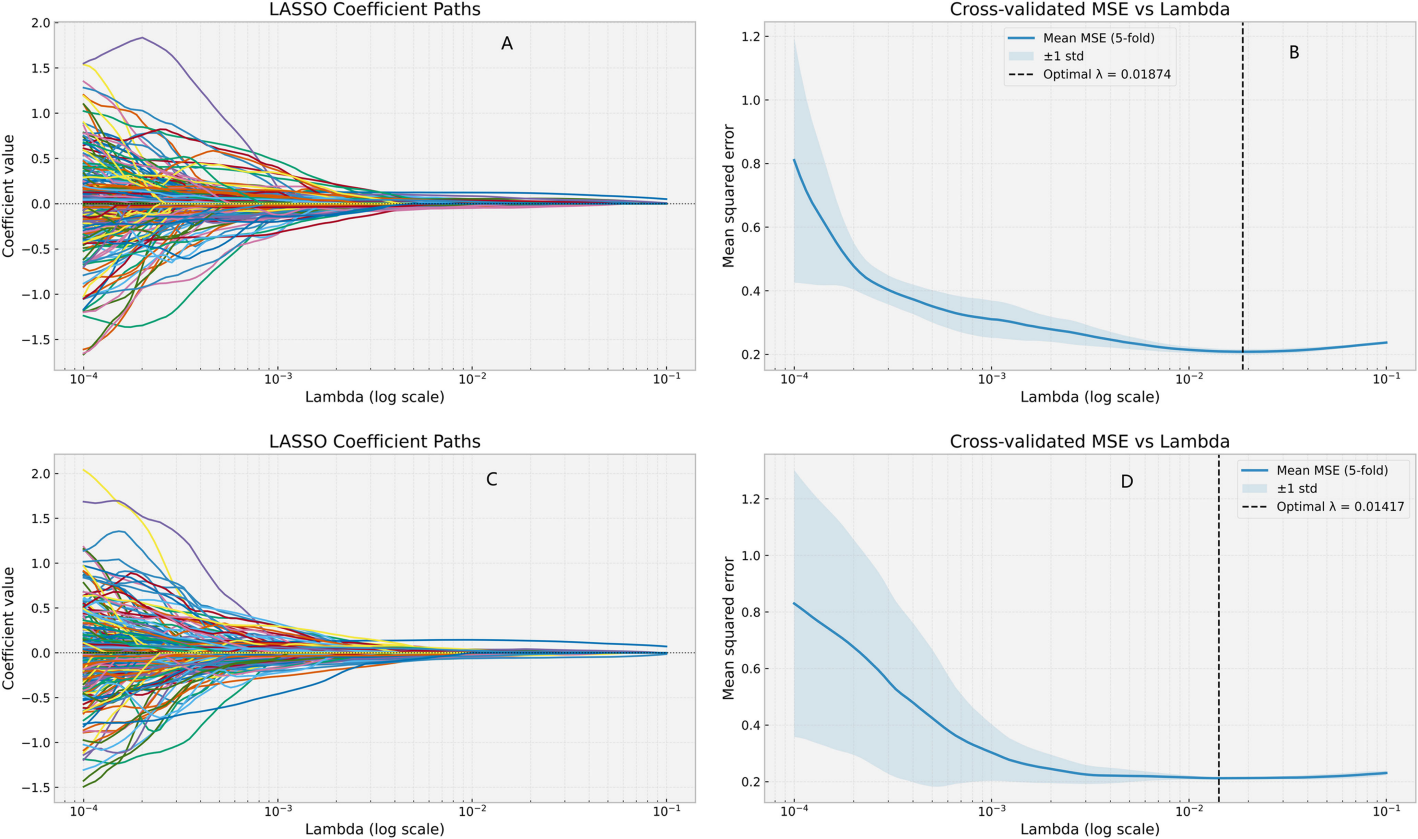
LASSO regression screening feature map. **(A, B)** is Clinical+Radiomics+Contrast parameter RF model in predicting LNM. **(C, D)** is Clinical+Radiomics+Contrast parameter RF model in predicting CLNM.

**Figure 7:**
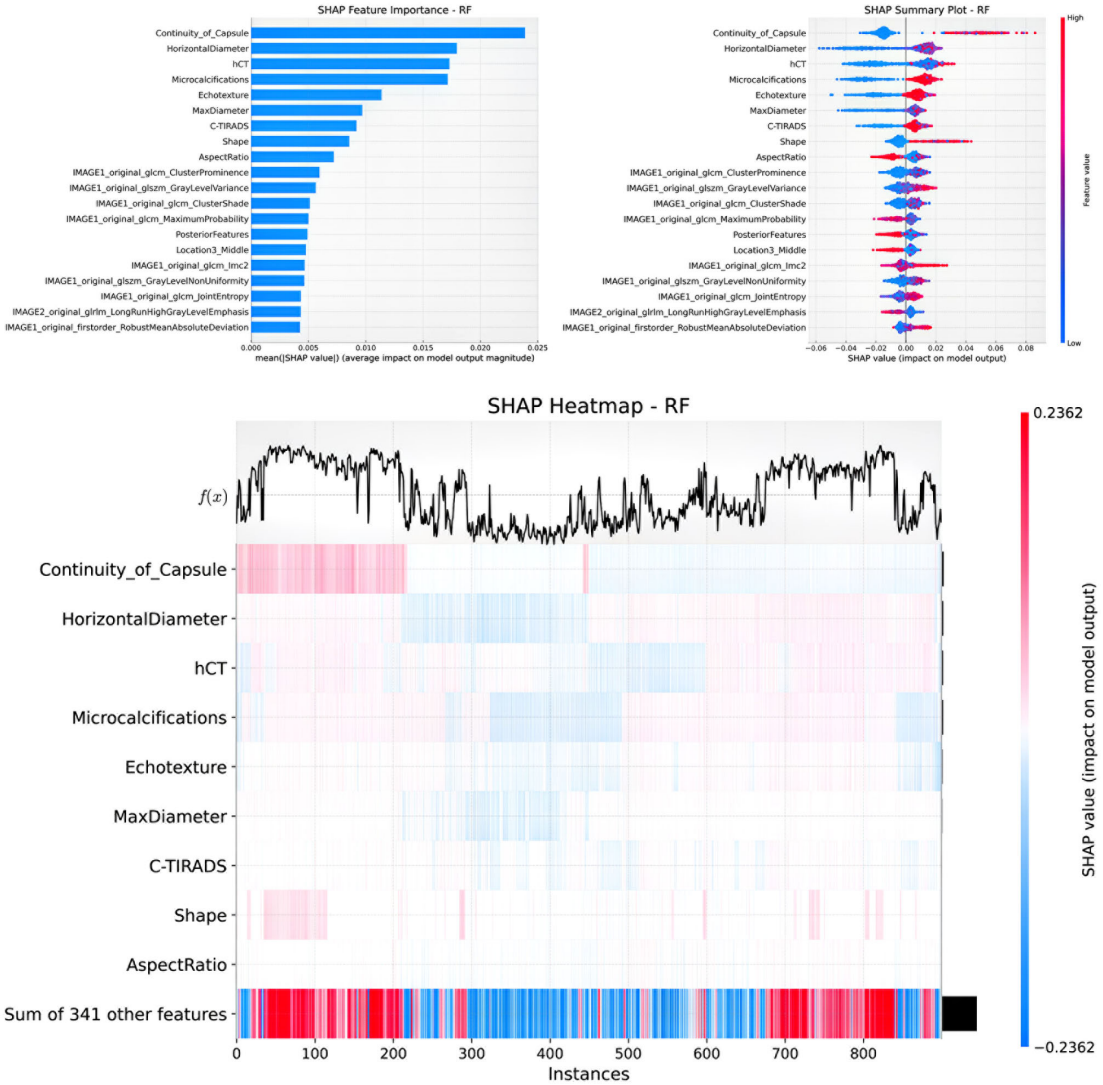
SHAP visualization analysis of clinical+radiomics+contrast parameter RF model in predicting CLNM.

**Figure 8:**
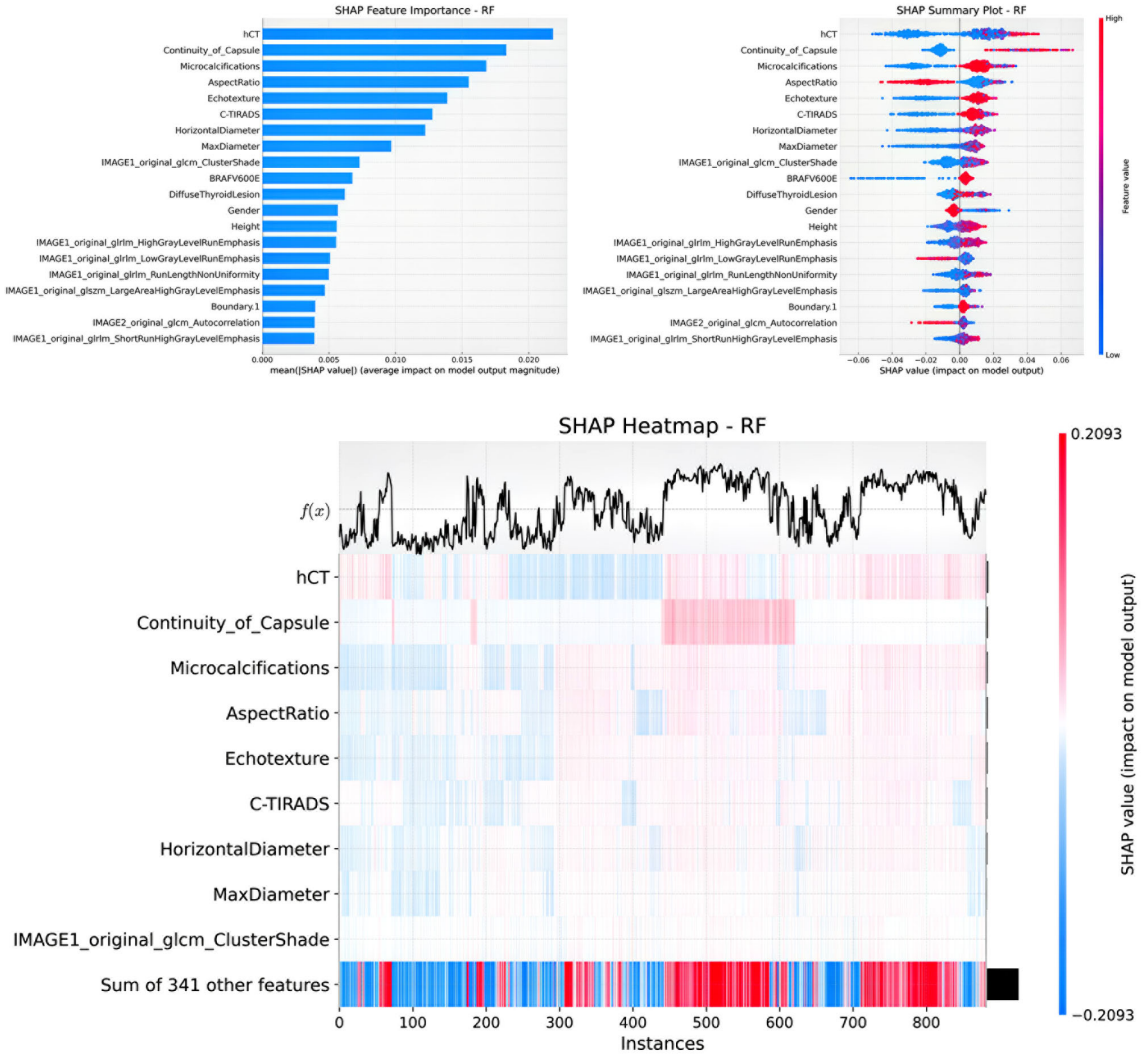
SHAP visualization analysis of clinical+radiomics+contrast parameter RF model in predicting LNM.

The LASSO regression analysis plot for the Clinical + Radiomics + Contrast Parameters RF model is presented in **Figure 6**, demonstrating the λ parameter selection and the relative importance of each predictive feature. SHAP values were applied to visualize the contribution of specific features to the model’s diagnostic outcomes. SHAP bar charts (**Figures 7** and **8**) display the mean absolute SHAP values for all features, allowing identification and ranking of the most influential predictors. The SHAP scatter plot illustrates the distribution of positive and negative impacts of each feature on the model’s predictions, with red indicating a positive influence and blue indicating a negative influence.

The SHAP heatmap presents feature contributions across individual cases, with rows corresponding to specific features and columns corresponding to patient instances. Cell colors indicate the magnitude and direction of each feature’s impact (red representing increased prediction probability, blue representing decreased probability, and white indicating no significant impact). The line chart at the top presents the output value f(x) for each instance, with the x-axis labeled by instance number and the y-axis scaled proportionally to the prediction range. This multi-dimensional visualization method provides an intuitive representation of prediction variability across samples and the correlation of SHAP values with prediction outcomes.

### CLNM prediction

The optimal model for CLNM prediction incorporated a total of 20 features, comprising 9 radiomics indicators, 10 clinical indicators, and 1 contrast parameter. Key features included capsule continuity, horizontal diameter, hCT, microcalcifications, echo texture, maximum diameter, TIRADS classification, tumor shape, aspect ratio, and various texture-based radiomics features (e.g., image1 original GLCM cluster prominence, image1 original GLSZM gray level variance, image1 original GLCM cluster shade, image1 original GLCM maximum probability, posterior features, tumor location, image1 original GLCM LMC2, image1 original GLSZM gray level nonuniformity, image1 original GLCM joint entropy, image2 original GLRLM long run high gray level emphasis, and image1 original first-order robust mean absolute deviation). Features with detrimental effects included aspect ratio, image1 original GLCM maximum probability, posterior features, tumor location, and image2 original GLRLM long run high gray level emphasis. Other features contributed positively to prediction performance.

### LNM prediction

The optimal model for LNM prediction incorporated 20 features, including 7 radiomics indicators, 11 clinical indicators, and 2 contrast parameters. Key features included hCT, capsule continuity, microcalcifications, aspect ratio, echo texture, TIRADS classification, horizontal diameter, maximum diameter, image1 original GLCM cluster shade, BRAFV600E mutation status, diffuse thyroid lesion, gender, height, image1 original GLRLM high gray level run emphasis, image1 original GLRLM low gray level run emphasis, image1 original GLRLM run length nonuniformity, image1 original GLSZM large area high gray level emphasis, boundary after enhancement, image2 original GLCM autocorrelation, and image1 original GLRLM short run high gray level emphasis. Negative effects were observed for aspect ratio, gender, image1 original GLRLM low gray level run emphasis, and image2 original GLCM autocorrelation, whereas other features contributed positively to prediction accuracy.

These SHAP-based analyses provide a detailed understanding of how individual clinical, radiomics, and contrast imaging parameters contribute to the predictive performance of the model for both CLNM and LNM, thereby supporting the identification of clinically relevant features for preoperative risk assessment.

## Discussion

PTC remains the most prevalent type of thyroid malignancy, with surgical intervention constituting the primary treatment (10). The 2015 American Thyroid Association (ATA) guidelines recommend therapeutic central lymph node dissection (CLND) in patients with LNM (11), whereas the role of prophylactic central lymph node dissection (pCND) in patients with cN0 remains contentious. This approach carries inherent risks, including recurrent laryngeal nerve injury, hypoparathyroidism, and chyle leakage (12). Several studies have reported that bilateral pCND followed by personalized radioiodine significantly improves ten-year disease-specific survival and local disease control in PTC with bilateral prophylactic central lymph node dissection(pCND) followed by personalized radioiodine therapy (13). However, nearly half of patients with PTC lack CLNM and thus do not derive benefit from routine prophylactic dissection, while being exposed to potential surgical complications. Notably, approximately 53.5% of patients with cN0 harbor occult CLNM (14), which may alter tumor-node-metastasis (TNM) staging and influence therapeutic decision-making for certain patients (15). In the present study, CLNM was observed in approximately 25.5% of patients and LNM in 26.9%, rates lower than those reported in prior studies, potentially attributable to enhanced diagnostic accuracy provided by CEUS. Nevertheless, precise preoperative identification of CLNM remains critically important for optimizing surgical planning and improving patient outcomes.

Comprehensive clinical investigations have been conducted to predict LNM based on clinical features and pathological classifications of papillary thyroid carcinoma (16). In addition, nomogram-based and machine learning models have been developed to predict metastatic status of cervical lymph nodes in PTC using ultrasound characteristics (17–19). He et al. (20) reported that a nomogram integrating CEUS predictive features demonstrated satisfactory discrimination and calibration in both training and validation cohorts, with AUC values of 0.72 and 0.79, respectively. However, there is a notable scarcity of established predictive models incorporating radiomics features extracted from ultrasound images. Specifically, radiomics models integrating CEUS imaging have been rarely investigated. Sun et al. (21) examined the value of a radiomics model combined with CEUS in predicting malignant thyroid nodules and found that the integration of radiomics features from the peritumoral area with CEUS significantly enhanced diagnostic performance.

In this study, radiomics features extracted from CEUS images were integrated into the model construction. Additionally, contrast parameters were incorporated into the radiomics model, resulting in a significant improvement in predictive performance. The findings demonstrated that the Clinical + Radiomics + Contrast parameter RF model exhibited the highest predictive accuracy for both CLNM and overall LNM. Notably, the optimal models for CLNM and LNM incorporated different predictive indicators, thereby offering the potential for more personalized diagnostic and treatment strategies tailored to the specific prediction requirements of individual patients.

In this study, the selected indicators in both optimal RF models included capsule continuity, horizontal diameter, hCT, microcalcifications, echo texture, maximum diameter, TIRADS classification, aspect ratio, and middle location. These predictors exhibited significant value in forecasting both CLNM and LNM. Huang et al. (22) identified microcalcifications as an important factor associated with CLNM in PTC, which aligns with the findings of the present study. The capsule continuity index also demonstrated high predictive value in this analysis. Zhang et al. (23) reported that assessment of extracapsular extension (ECE) is clinically relevant for predicting cervical LNM, a conclusion consistent with the present results. Tumor size has been recognized as an independent risk factor for CLNM in PTC (24, 25). In the current study, horizontal diameter and maximum diameter emerged as key predictors of LNM, consistent with the observation that larger tumor size is associated with a higher likelihood of metastasis.

Noel et al. evaluated the association between thyroglobulin antibody (TG-Ab) levels and LNM in patients with differentiated thyroid carcinoma and found that preoperative TG-Ab levels served as an independent predictor of LNM (26). Additionally, TSH has been confirmed as a growth factor influencing the occurrence and progression of PTC (27). In the present study, hCT levels demonstrated a positive correlation with the occurrence of CLNM, further supporting the value of tumor markers in predicting lymph node metastasis.

Each lobe of the thyroid contains its own internal lymphatic system (28) and metastasis generally follows a pattern from the central compartment to the ipsilateral cervical region, subsequently extending to contralateral and distant lymph nodes. Tumor location in the upper lobe and PTC have been identified as a risk factor for skip metastasis (29). In the present study, tumor location in the middle lobe emerged as an important predictor, potentially related to lymphatic drainage pathway. Further studies are warranted to confirm this correlation. Additionally, gender was found to be a significant predictor in LNM, with 27% of female patients and 41.1% of male patients exhibiting metastasis. The likelihood of cervical lymph node metastasis was markedly higher in male patients compared to female patients.

The BRAF gene mutation is widely recognized as a critical factor in the development and progression of PTC. Previous studies have reported that patients harboring BRAF mutations often exhibit aggressive pathological characteristics (30). This mutation is associated with elevated expression of oncogenic factors, including vascular endothelial growth factor (VEGF) and metalloproteinases (MET). However, its role as a prognostic marker remains debated due to limited specificity in predicting disease recurrence (31). In the present study, the BRAFV600E mutation was identified as a predictor of LNM, but not CLNM, thereby underscoring its potential value in predicting cervical lymph node involvement.

Artificial intelligence (AI)–based radiomics, enables the extraction of high-throughput quantitative features from imaging data to reveal underlying disease characteristics (9). Radiomics analysis methods facilitate the decoding of high-dimensional tumor imaging phenotypes from specific areas (32), and have been applied to distinguish cervical LNM status in PTC. However, the predictive performance of radiomics-based approaches for LNM has remained suboptimal, with AUC values ranging from 0.727 to 0.803 (33–35). In the present study, the AUCs of the two optimal models were 0.928 and 0.942, substantially exceeding those reported for conventional prediction models. Radiomics features were found to play a significant role in predicting lymph node metastasis. Furthermore, incorporating CEUS images introduced additional important predictors, including original glrlm long-run high gray level emphasis and original glcm autocorrelation, along with conventional ultrasound radiomics features.

Ultrasound is a convenient, safe, and cost-effective imaging modality and remains the primary choice for evaluating thyroid cancer. Consequently, a prognostic model based on ultrasound images may offer greater practicality and accuracy over time. CEUS provides detailed insight into lesion perfusion patterns and has been reported as an effective imaging technique for differentiating malignant from benign lymph nodes and for identifying metastatic cervical lymph nodes in patients with PTC (36).

In this study, contrast parameter indices were incorporated, and the results demonstrated that these parameters contributed to the construction of both prediction models. Specifically, one contrast parameter was involved in predicting CLNM, while two parameters were involved in predicting LNM. Feature selection further confirmed the predictive value of contrast parameters, including capsule continuity and boundary after enhancement, in both models.

Some studies have indicated that incorporating superb microvascular imaging (SMI) in prediction models achieves diagnostic performance similar to that of CEUS for identifying malignant thyroid nodules (37). However, PTC frequently exhibits poor vascularization on color Doppler flow imaging (CDFI); in this study, 439 patients (72.7% of the cohort) demonstrated no detectable blood flow signal on CDFI. This indicates that radiomics models relying solely on blood flow imaging may have limitations for such patients. As a diagnostic tool, CEUS is recognized for its ability to assess microvascularization, which is critical given that angiogenesis is fundamental to neoplastic development (38). Therefore, for predicting LNM in patients with PTC with poor vascular supply, the prediction model developed in this study may demonstrate superior performance, a possibility that warrants further validation in future research.

In this study, five commonly used machine learning models for evaluation were selected. Among these, the RF model demonstrated the highest predictive performance, consistent with the findings of Chen, who reported superior diagnostic efficacy of RF in differentiating benign and malignant thyroid nodules classified as TIRADS 4a using ultrasound angiography (39). SVM is a supervised learning-based binary generalized linear classifier, particularly suited for addressing complex nonlinear problems compared with LR analysis. LR analysis is applicable for multiclass classification tasks and performs well in scenarios with smaller datasets. RF, an extension of parallel ensemble learning, introduces randomness in the selection of partition attributes, thereby enhancing model robustness. GBM is an ensemble algorithm that combines multiple weak learners to improve predictive performance, while XGBoost further supports custom loss functions, additional regularization, handling of missing values, and column sampling (40). Among these models, RF exhibited superior convergence to lower generalization error. Additionally, RF selects partition attributes from a subset of available features, improving training efficiency. Its performance is more stable, parameter tuning is relatively simpler, computational time is shorter, and its applicability across different datasets is stronger.

This model demonstrates the capability to predict early LNM in PTC, thereby enabling more timely and effective treatment interventions, reducing the risk of postoperative complications associated with unnecessary central lymph node dissection, and supporting the development of personalized treatment strategies for patients.

Although the radiomics model achieved a high AUC value (0.928-0.942) and high specificity, the primary limitation of this study was its insufficient sensitivity. This low sensitivity suggests a potentially high false-negative rate. This phenomenon is mainly driven by category imbalance in the training cohort and the use of default probability thresholds, which lead the model to prioritize specificity. While high specificity ensures the high reliability of positive predictions—potentially aiding in the decision-making for therapeutic neck dissection—the clinical priority for managing metastatic lymph nodes often requires minimizing missed diagnoses. Therefore, the current clinical value of this model warrants further investigation. Future studies should explore alternative decision thresholds or implement cost-sensitive learning strategies to significantly reduce the false-negative rate, thereby achieving a more clinically feasible balance between sensitivity and specificity.

However, this study has several limitations. First, the single-center retrospective design lacks external validation, rendering the generalizability of the current model unknown. Consequently, these findings should be interpreted cautiously, necessitating future prospective, multi-center cohorts to rigorously evaluate their true robustness and clinical applicability. Second, methodological constraints regarding image acquisition and formatting may affect feature robustness. The reliance on lossy JPEG images rather than uncompressed DICOM data introduces potential compression artifacts that inherently compromise radiomic fidelity. Compounding this issue, images were acquired across three ultrasound systems without applying feature harmonization techniques. This multi-vendor heterogeneity likely introduced batch effects, underscoring the strict necessity of utilizing original DICOM data and robust harmonization algorithms in future multi-center studies. Third, the lack of rigorous reproducibility and quantitative assessments introduces potential observer bias. Manual ROI delineation was performed by a single physician without intraclass correlation coefficient (ICC) evaluation, leaving the stability of extracted features unverified. Similarly, CEUS evaluation was entirely qualitative and operator-dependent, as the retrospective design precluded objective time-intensity curve (TIC) analysis. Furthermore, our radiomic features were derived solely from static images; exploring dynamic video data could capture more comprehensive tumor hemodynamics. Future prospective designs must mandate multi-observer segmentations, quantitative TIC extraction, and dynamic imaging analysis to fully standardize these metrics.

## Conclusion

In this study, we constructed a prediction model integrating CEUS images and clinical features. Twenty clinical and radiomics indicators were selected to develop a RF model, with their contributions visualized using the SHAP method to account for synergistic interactions. The findings indicated that incorporating radiomics and CEUS contrast parameters improved the AUC of the model for predicting both LNM and CLNM.

However, while the integration of clinical, radiomics, and contrast parameters achieved high specificity, its clinical utility is currently severely limited by low sensitivity and the absence of external validation. Therefore, rather than providing a definitive clinical tool, our findings should be viewed strictly as a preliminary exploratory study. The true clinical applicability of these predictive signatures remains to be established through future prospective, multi-center studies designed (41).

## Data Availability

The original contributions presented in the study are included in the article/Supplementary Material. Further inquiries can be directed to the corresponding author.

## Glossary

CEUS: contrast-enhanced ultrasound
PTC: papillary thyroid carcinoma
LNM: lymph node metastasis
CLNM: central lymph node metastasis
cN0: clinical no regional lymph node metastasis
RF: random forest
ROC: receiver operating characteristic
PR: precision-recall
SHAP: SHapley Additive exPlanations
AUC: area under curve
LNs: lymph nodes
US: ultrasound
AI: Artificial intelligence
SWE: Shear wave elastography
JPG: joint photographic experts group
NII: neuroimaging informatics technology initiative
ROI: Regions of interest
XGBoost: extreme gradient boosting
GBM: gradient boosting machine
SVM: support vector machine
LR: logistic regression
BMI: body mass index
Emax: elastography maximum value
Emean: elastography mean value
Emin: elastography minimum value
EI: elastography ratio
TIRADS: Thyroid Imaging Reporting and Data System
TSH: thyroid stimulating hormone
FT4: free thyroxine
FT3: free triiodothyronine
Anti-TG: thyroglobulin antibodies
Anti-TM: thrombomodulin antibody
Tg: thyroid protein
PTH: parathormone
hCT: hematocrit
DCA: decision curve analysis
ATA: American Thyroid Association
CLND: central lymph node dissection
pCND: prophylactic central lymph node dissection
TNM: tumor node metastasis
ECE: extracapsular extension
TG-Ab: thyroglobulin antibody
DTC: differentiated thyroid carcinoma
VEGF: vascular endothelial growth factor
MET: metalloproteinase
SMI: superb microvascular imaging
CDFI: color doppler flow imaging
PR_AP: precision recall average precision.
